# Single nucleotide polymorphisms in surfactant protein A1 are not associated with a lack of responsiveness to antenatal steroid therapy in a pregnant sheep model

**DOI:** 10.14814/phy2.15477

**Published:** 2022-10-05

**Authors:** Tsukasa Takahashi, Yuki Takahashi, Erin L. Fee, Haruo Usuda, Lucy Furfaro, John P. Newnham, Alan H. Jobe, Matthew W. Kemp

**Affiliations:** ^1^ Division of Obstetrics and Gynaecology The University of Western Australia Perth Western Australia Australia; ^2^ Centre for Perinatal and Neonatal Medicine Tohoku University Hospital Sendai Japan; ^3^ Perinatal Research, Department of Pediatrics Cincinnati Children's Hospital Medical Centre, University of Cincinnati Cincinnati Ohio USA; ^4^ School of Veterinary and Life Sciences Murdoch University Perth Western Australia Australia; ^5^ Department of Obstetrics and Gynaecology Yong Loo Lin School of Medicine, National University of Singapore Singapore

**Keywords:** antenatal corticosteroids, lung maturation, preterm birth, sheep, single nucleotide polymorphisms

## Abstract

Treatment with antenatal steroids (ANS) is standard practice for reducing the risk of respiratory distress in the preterm infant. Despite clear overall benefits when appropriately administered, many fetuses fail to derive benefit from ANS therapies. In standardized experiments using a pregnant sheep model, we have demonstrated that around 40% of ANS‐exposed lambs did not have functional lung maturation significantly different from that of saline‐treated controls. Surfactant protein A is known to play an important role in lung function. In this genotyping study, we investigated the potential correlation between polymorphisms in *SFTPA1*, messenger RNA and protein levels, and ventilation outcomes in animals treated with ANS. 45 preterm lambs were delivered 48 h after initial ANS therapy and 44 lambs were delivered 8 days after initial ANS therapy. The lambs were ventilated for 30 min after delivery. *SFTPA1* mRNA expression in lung tissue was not correlated with arterial blood PaCO_2_ values at 30 min of ventilation in lambs delivered 48 h after treatment. SFTPA1 protein in lung tissue was significantly correlated with PaCO_2_ at 30 min of ventilation in lambs ventilated both 48 h and 8 days after ANS treatment. Six different single nucleotide polymorphisms (SNPs) in the *Ovis aries SFTPA1* sequence were detected by Sanger Sequencing. No individual SNPs or SNP haplotypes correlated with alterations in PaCO_2_ at 30 min of ventilation or SFTPA1 protein levels in the lung. For the subset of animals analyzed in the present study, variable lung maturation responses to ANS therapy were not associated with mutations in *SFTPA1*.

## Graphical abstract

In the sheep pregnant model, fetal lung SP‐A protein concentration but not mRNA expression was correlated to the fetal lung maturation after ANS treatment. However, none of the SNPs in the SP‐A gene were associated with fetal lung maturation or variability of ANS treatment responsiveness.

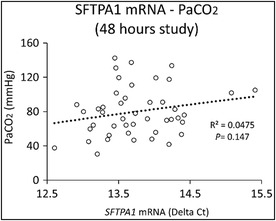

## 1 BACKGROUND

Antenatal steroid (ANS) therapy is a standard treatment for women at risk of imminent preterm delivery (Antenatal Corticosteroid Therapy for Fetal Maturation, 2017; World Health Organization, 2015). ANS therapy improves perinatal outcomes by promoting fetal lung maturation, reducing the risk and severity of respiratory distress syndrome (RDS) and intraventricular hemorrhage (Liggins & Howie, 1972; McGoldrick et al., 2020). Not all fetuses derive benefits from ANS therapy (Takahashi, Jobe, et al., 2022). Some of this non‐responsiveness relates to off‐target treatments; it is estimated that as many as half of all fetuses exposed to ANS treatment remain undelivered 7 days after treatment, the point at which therapeutic benefit is significantly reduced or lost (Adams et al., 2015; Jobe & Goldenberg, 2018; Makhija et al., 2016; McLaughlin et al., 2003). According to a recent Cochrane review, the number needed to treat to prevent one case of RDS at 24–35 weeks of gestation is 19 (McGoldrick et al., 2020). As such, even on‐target ANS use is associated with a sizable amount of residual morbidity.

Our recent animal studies, employing a well‐standardized pregnant sheep model and post‐natal ventilation, allowed us to assess ANS responses whilst minimizing experimental noise inherent to clinical studies, including variable treatment to delivery interval, drug dose and type, co‐morbidity (i.e. chorioamnionitis), environmental effects (smoking, alcohol), diet, and differing clinical assessments. In our model system, around 40% of the lambs routinely fail to respond to maternal ANS therapy in terms of fetal lung maturation (Takahashi et al., 2020). We previously found that total betamethasone concentrations or unbound betamethasone concentrations in fetal and maternal plasma were not associated with responsiveness to ANS therapy (Takahashi et al., 2020). These data suggest that fetus‐specific factors may influence ANS responsiveness. Given the correlation between SPA concentrations and maturation outcomes, we hypothesized that single nucleotide polymorphisms (SNPs) in surfactant protein A may explain some of the individual variations in responsiveness to ANS therapy.

## 2 METHODS

### 2.1 Animal work

This study is a secondary analysis of previously reported animal studies (Takahashi et al., 2021; Takahashi, Takahashi, et al., 2022). All protocols were reviewed and approved by the animal ethics committee of The University of Western Australia (RA/3/100/1636, RA/3/100/1702) and Murdoch University (R3330/21). All pregnant ewes were sourced from a single supplier and experiments were performed during the normal breeding season over the winter months. A total of 150 mg of medroxyprogesterone acetate (Depo‐Ralovera®; Pfizer, West Ryde, NSW, Australia) was administered to each ewe 5 days prior to steroid treatment commencing to prevent preterm labor. This treatment does not impact the maturation of the fetal ovine lung (Jobe et al., 2003). Two groups of animals were analyzed in this study, with surfactant protein A and ventilation data (other than for surfactant protein A in dexamethasone‐treated animals) as published previously (Takahashi et al., 2021; Takahashi, Takahashi, et al., 2022). Twelve singleton pregnant ewes were assigned to the Saline Control Group and received four maternal intramuscular saline injections at 115, 116, 117, and 118 days' gestation before lambs were delivered at 123 days' gestation. This group was used as the control group for both the 2‐day and eight‐day treatment to delivery interval studies, in keeping with good ethical practice to minimize animal numbers.

### 2.2 Two‐day treatment to delivery interval

Forty‐five steroid‐treated animals were analyzed from this group. Animals were assigned at random to one of four groups: (i) a single course Betamethasone Phosphate+Acetate (Beta‐P + Ac) Group receiving two maternal intramuscular injections of 0.25 mg/kg Beta‐P + Ac spaced by 24 h on 121 and 122 days' gestation (*n* = 12); (ii) a single course Betamethasone Acetate (Beta‐Ac) Group receiving two maternal intramuscular injections of 0.125 mg/kg Beta‐Ac only (Hovione, Portugal) spaced by 24 h on 122 and 123 days' gestation (*n* = 11); or (iii) a single course Dexamethasone Phosphate (Dex‐P) Group receiving four maternal intramuscular injections of 6 mg Dex‐P (DBL Dexamethasone sodium phosphate 4 mg/ml, Hospira NZ, New Zealand) at 12 h intervals on 122 ± 1 days' gestation (*n* = 22). Then lambs were delivered 48 h after initial treatment under terminal anesthesia and ventilated for 30 min.

### 2.3 Eight‐day treatment to delivery interval

Forty‐four animals were analyzed in this group. Animals were assigned at random to one of four groups: (i) a d8,7,6 Beta‐Ac Group, which received three maternal intramuscular injections of 0.125 mg/kg Beta‐Ac on days 8, 7, and 6 prior to delivery (*n* = 11); (ii) a d8,7,6,5 Beta‐Ac Group, which received four maternal intramuscular injections of 0.125 mg/kg Beta‐Ac on days 8, 7, 6 and 5 prior to delivery (*n* = 11); (iii) a d8,6 pulsed Beta‐P + Ac Group, which received two maternal intramuscular injections of 0.25 mg/kg Beta‐P + Ac on days 8 and 6 prior to delivery (*n* = 10); or (iv) a d8,6 pulsed Beta‐Ac Group, which received two maternal intramuscular injections of 0.125 mg/kg Beta‐Ac on days 8 and 6 prior to delivery (*n* = 12). Lambs were delivered at 121–123 days' gestation under terminal anesthesia and immediately ventilated for 30 min.

### 2.4 Delivery, ventilation, and necropsy

Delivery and ventilation methods were as previously reported (Takahashi et al., 2020, 2021). Briefly, lambs were surgically delivered and had a tracheostomy to install a secured endotracheal tube. Lambs were then weighed, quickly dried, and ventilated for 30 min, initially with the following parameters using heated and humidified 100% oxygen: a maximal peak inspiratory pressure (PIP) of 35 cmH_2_O, a positive end‐expiratory pressure (PEEP) of 5 cmH_2_O, a respiratory rate of 50 breaths per minute, an inspiratory time of 0.5 s. Tidal volume (Vt) was kept between 7.0 and 8.0 ml/kg by adjusting PIP. A single umbilical artery catheter was inserted to allow measurement of arterial blood pH, PaO_2_, PaCO_2_, heart rate, and blood pressure. Lambs were euthanized after 30 min of ventilation and promptly subjected to necropsy. Fetal lung tissue was collected and frozen as previously described (Takahashi et al., 2020).

### 2.5 Measurement of RNA transcript expression changes in the fetal lung

Messenger ribonucleic acid (mRNA) was extracted from fetal lung tissue (right lower lobe) using a RNeasy Plus Mini kit (QIAGEN) according to the manufacturer's instructions. The concentration of extracted mRNA was determined by a broad‐range nucleic quantitation kit and a Qubit 2.0 Fluorometer (both Life Technologies). All mRNA extracts were diluted in nuclease‐free water (Life Technologies) to achieve a final concentration of 25 ng/μl. Quantitative polymerase chain reaction (qPCR) cycling was performed with ovine‐specific TaqMan probe and primer sets (Applied Biosystems) on an OneStep Real‐Time PCR System according to the manufacturer's instructions. Surfactant protein A (*SP‐A*) was measured using 18 s ribosomal protein as internal reference to normalize the amplification data. Delta quantification cycle values were used to determine to relative expression of transcripts.

### 2.6 Protein quantification by Western blot assay

Relative amounts of SFTPA1 protein in the lung tissue were measured by fluorescent western blot assay. 20 μg of reduced protein was used for SFTPA1 measurements. Protein electrophoresis and transfer was as published previously (Takahashi et al., 2021). Once proteins were transferred to PVDF membranes, they were incubated with primary antibody overnight at 4°C. Primary antibodies, anti‐surfactant protein A/PSAP antibody (ab115791, abcam), were diluted into SuperSignal Western Blot Enhancer (Thermo Scientific) at 1/1000. Washed membranes were incubated with Goat anti‐Rabbit IgG (H + L) Highly Cross‐Adsorbed Secondary Antibody, Alexa Fluor Plus 800 (Invitrogen) at 1/10,000 in wash buffer (phosphate‐buffered saline with 0.05% Tween 20; both Sigma‐Aldrich.) for 60 min. Membranes were analyzed using an iBright FL1000 Imaging System (Invitrogen) and target band concentrations were measured and normalized by total protein concentration. In order to normalize the difference between membranes, standard quality control samples were transferred to each membrane and probed.

### 2.7 Genotyping by sanger sequencing

**FIGURE 1:**
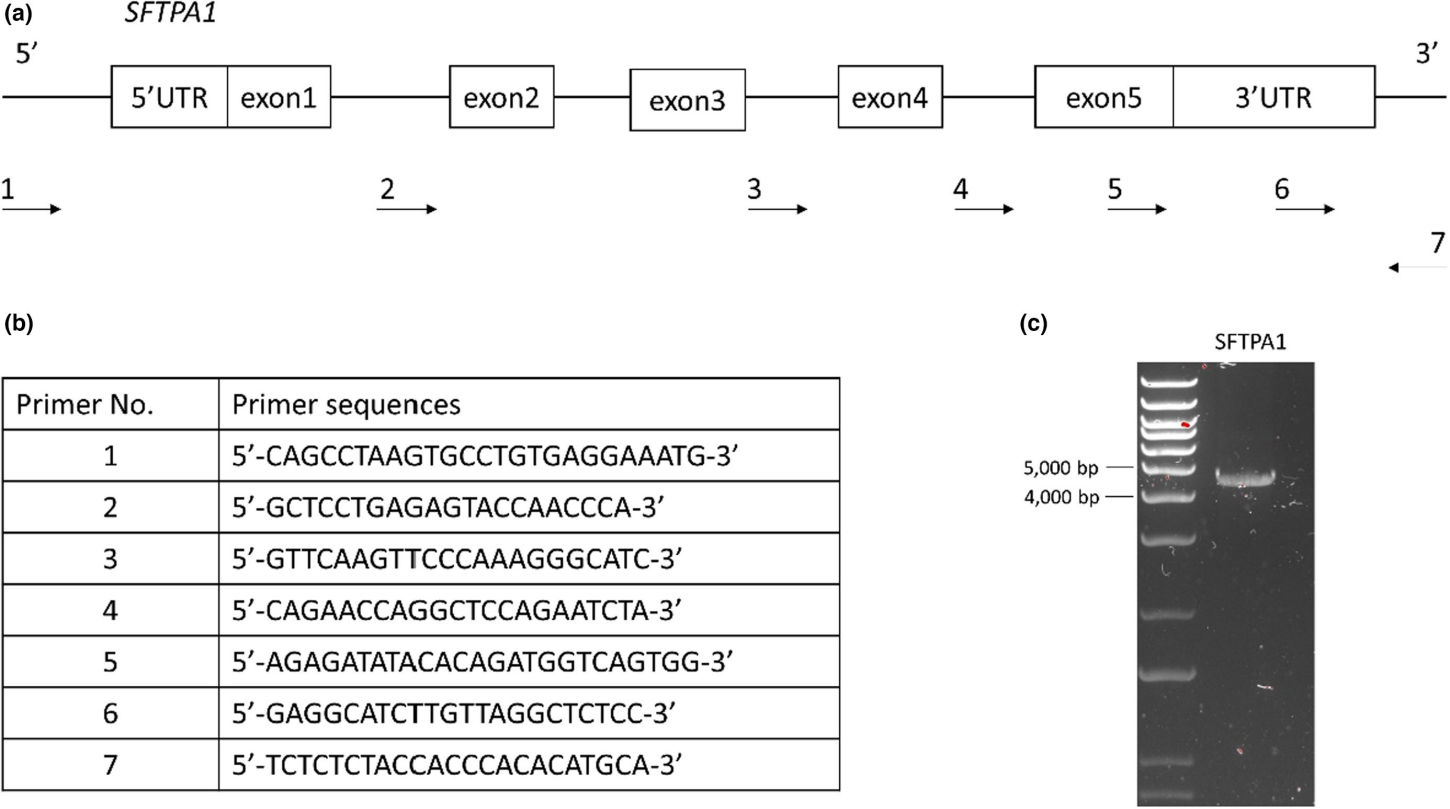
Map of primers. (a, b) Ovis *SFTPA1* consists of five exons with 5′ and 3′ UTRs. Primer 1 and primer 7 were set as forward and reverse primers respectively to include all SFTPA1 sequences. Then Primer 1–6 were used for Sanger Sequencing. (c) PCR product using primer 1 and 7 was at 4.4 kbp.

DNA samples were extracted from fetal lung tissue using a DNeasy® Blood & Tissue Kit (QIAGEN) according to the manufacturer's instructions. Forward and reverse primers were designed using the *Ovis aries SFTPA1* sequence (NCBI; NM_001009728.2). The amplified region included the entire *SFTPA1* sequence (Figure 1a,b). 5 μl of DNA of samples were used for PCR using a UltraRange Long PCR kit (QIAGEN) in accordance with the manufacturer's protocol. Amplified samples were separated in a 1.0% agarose gel and at 130 V for 30 min. The predicted amplicon size for SFTPA1 was 4.4 kbp (Figure 1c). The target band was excised from the gel using an iBright FL1000 Imaging System. The samples were cleaned using a QIAquick Gel Extraction Kit (QIAGEN) and DNA concentration was measured using a Qubit 2.0 Fluorometer (Life Technologies).

Amplicon samples were submitted along with 6 different forward primers to the Australian Genome Research Facility for Sanger sequencing (Figure 1b). Each sample *SFTPA1* sequence was compared to *SFTPA1* sequence (NM_001009728.2) to detect SNPs with alignments performed using Unipro UGENE version 37.0. Polymorphisms, haplotypes, and diplotypes for fetal *SFTPA1* were then determined.

### 2.8 Genotyping and lung maturation analysis

A total of 89 animals were sequenced. Each SNP, haplotype, and diplotype was compared to 30 min ventilation PaCO_2_ values and fetal lung SFTPA1 protein concentrations. The comparison between each SNP and PaCO_2_ in the d8,6 pulsed Beta‐P + Ac and d8,6 pulsed Beta‐Ac groups was eliminated because PaCO_2_ was not improved, compared to the saline control group (Table 1). Since SFTPA1 was increased in all groups compared to the saline control group, all groups were used for comparison between SNPs and SFTPA1 protein.

**TABLE 1:** PaCO_2_ and SFTPA1 protein amount in each group

	*n*	PaCO_2_ (mmHg)	SFTPA1 protein fold change (vs. control)
Saline control group	12	118 ± 21	1.0 ± 0.3
2 days study			
Beta‐P + Ac group	12	82 ± 24^a^	1.8 ± 0.7^a^
Beta‐Ac group	11	64 ± 15^a^	2.4 ± 0.7^a^
Dex group	22	87 ± 31^a^	2.1 ± 1.0^a^
8 days study			
d8,7,6 Beta‐Ac group	11	72 ± 35^a^	2.3 ± 0.7^a^
d8,7,6,5 Beta‐Ac group	11	57 ± 24^a^	2.2 ± 0.6^a^
d8,6 pulsed Beta‐P + Ac group	10	82 ± 50	2.7 ± 1.4^a^
d8,6 pulsed Beta‐Ac group	12	109 ± 24	1.9 ± 0.6^a^

*Note*: These data were reported previously (Takahashi et al., 2021; Takahashi, Takahashi, et al., 2022b).

^a^ There were significant differences compared to the Saline Control Group. (*p* < 0.05).

### 2.9 Statistical analysis

Statistical analyses were performed using IBM SPSS Statistics for Windows, version 28.0 (IBM Corp). For correlation analyses between either *SFTPA1* mRNA or SFTPA1 protein and PaCO_2_ at 30 min of ventilation, delta Ct in mRNA or fold changes against a saline control group in protein were converted to a logarithmic scale and used. One‐way analysis of variance or *t*‐tests were used for comparing SNPs, haplotypes, or diplotypes.

## 3 RESULTS

### 3.1 Two‐day studies

**FIGURE 2:**
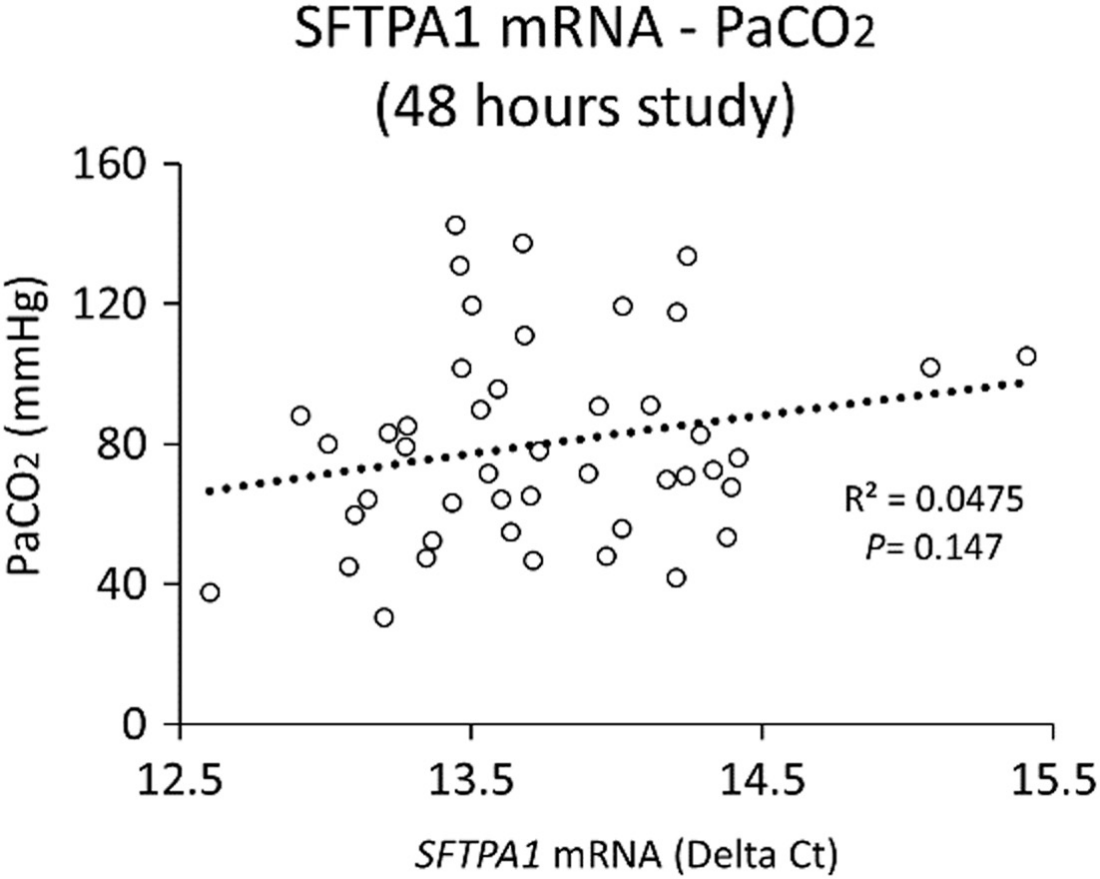
Correlations between SFTPA1 mRNA and lung function (48 h study only). The graph shows the correlation between *SFTPA1* mRNA expression and PaCO_2_ in two‐day study animals. There was no significant correlation between these values. (*p* = 0.147).

**FIGURE 3:**
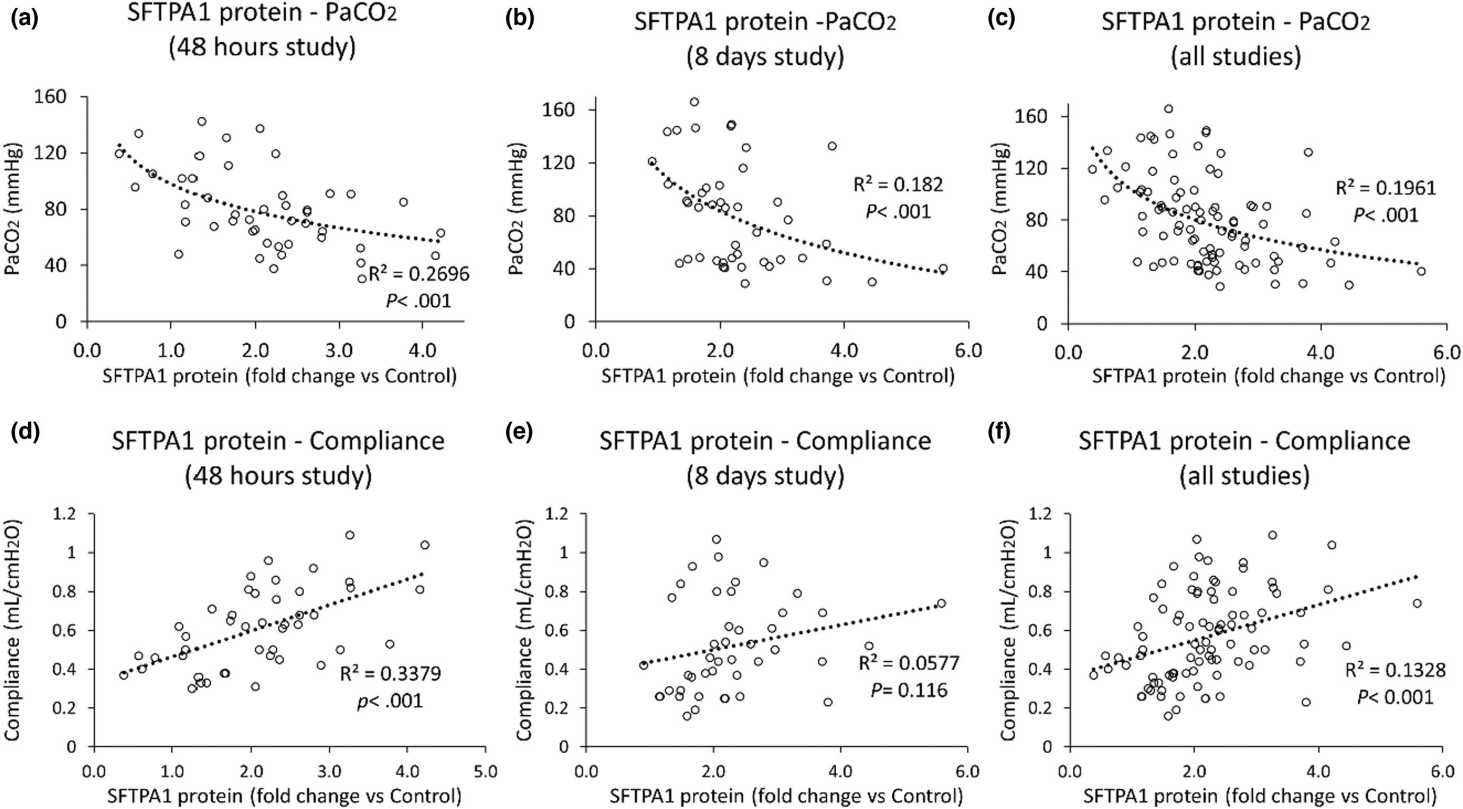
Correlation between SFTPA1 protein and lung function. (a–c) The graphs show correlations between SFTPA1 protein fold change against saline control group and PaCO_2_ in 48 h study, 8 days study, and all animals from 48 h and 8 days studies, respectively. Each graph shows a significant correlation between them (*p* < 0.001). (d–f) The graphs show a correlation between SFTPA1 protein fold change against a saline control group and lung compliance at 30 min ventilation in 48 h study, 8 days study, and all animals from 48 h and 8 days studies, respectively. While animals in 48 h study (d) and all animals (f) showed a significant correlation between them (*p* < 0.001), animals in 8 days study (e) did not (*p* = 0.116).

As shown previously, both Beta‐P + Ac, Beta‐Ac, and Dex treatment improved fetal lung maturation compared to the saline control groups at 48 h from initial treatment (Table 1) (Takahashi et al., 2021; Usuda et al., 2022). SFTPA1 protein was significantly increased in treated groups compared to the saline control groups (Table 1). mRNA expression of *SFTPA1* in treated animals was not correlated to PaCO_2_ at 30 min ventilation (*r* = 0.22, *p* = 0.147) (Figure 2). However, SFTPA1 protein concentration was correlated to PaCO_2_ (*r* = −0.52, *p* < 0.001) and lung compliance (*r* = 0.58, *p* < 0.001) at 30 min ventilation, respectively (Figure 3a,d).

### 3.2 Eight‐day studies

As previously shown, PaCO_2_ was significantly lower in the d8,7,6 Beta‐Ac and d8,7,6,5 Beta‐Ac groups than in the saline control group (Table 1) (Takahashi, Takahashi, et al., 2022). SFTPA1 protein was significantly increased in all groups compared to the saline control group (Table 1). Protein level of SFTPA1 was negatively correlated to PaCO_2_ at 30 min ventilation (*r* = −0.47, *p* < 0.001), but not to lung compliance (*r* = 0.24, *p* = 0.116) (Figure 3b,e). When the animals in 24 h and 8 days studies were combined, of SFTPA1 protein concentration remained significantly correlated to PaCO_2_ (*r* = −0.44, *p* < 0.001) and lung compliance (*r* = 0.37, *p* < 0.001) at 30 min ventilation (Figure 3c,f).

### 3.3 Genotyping by sanger sequencing

We detected 6 SNPs in *Ovis arrays SFTPA1*. Data are shown, along with the relevant number of animals represented with each allele, in Table 2. Description of sequence changes referred to the previous report (den Dunnen & Antonarakis, 2001).

**TABLE 2:** SNPs in ovis *SFTPA1*

	Coding DNA	Protein	Part of gene	Allele (number of animals)			
SNP1	c.2C > T	*p.(=)*	Exon 1	CC (1)	CT (21)	TT (67)	
SNP2	c.96C > T	*p.(=)*	Exon 2	CC (69)	CT (20)	TT (0)	
SNP3	c.107_108TG > CA (c.107 T > C)	p.Met36Thr (*p.(=)*)	Exon 2	TG (1)	TG/CT (20)	CA (65)	CA/CG (3)
SNP4	c.*338A > T	NA	3′UTR	AA (11)	AT (40)	TT (38)	
SNP5	c.*798C > T	NA	3′UTR	CC (69)	CT (20)	TT (0)	
SNP6	c.*880_*883del	NA	3′UTR	TTAT/TTAT (67)	TTAT/del (21)	del/del (1)	

*Note*: An * means the nucleotide located in the 3′UTR and the number is counted from the termination codon (den Dunnen & Antonarakis, 2001). *p.(=)* means silent changes (den Dunnen & Antonarakis, 2001).

SNP1 is c.2C > T at the exon 1 start codon. Although ACG is usually translated into threonine and the start codon should be ATG, according to NCBI, it is translated into methionine which is the start codon as previously reported (Peabody, 1987).

SNP2, which was c.96C > T, was a silent mutation at exon 2 and SNP3 was a missense mutation at the exon 2 from methionine to threonine (p.Met36Thr). Only three animals had CG instead of TG (c.107 T > C) which was a silent mutation and translated into methionine. SNP4, 5, and 6 were in the 3′UTR.

Five different haplotypes were detected and labeled from A to E (Table 3). In total 178 chromosomes (89 lambs), each haplotype accounts for following: A 12.4%, B 9.6%–11.2%, C 63.5%–65.2%, D 11.2%, E 1.7%. 75 out of 89 lambs had haplotype C at least once. Haplotype E was rare and found in only three lambs. We could not determine alleles precisely at SNP4 in haplotype E due to the small sample number. With regard to diplotype distribution, diplotype CC was most commonly identified, being present in 38 out of 89 lambs.

**TABLE 3:** Haplotypes and diplotypes of ovis *SFTPA1*

Haplotype	SNP1	SNP2	SNP3	SNP4	SNP5	SNP6	*n*
A	C	C	TG	A	C	del.	21
B	T	C	CA	A	C	TTAT	15
C	T	C	CA	T	C	TTAT	75
D	T	T	CA	A	T	TTAT	20
E	C	C	CG	A/T	T	TTAT	3

Diplotypes	AA	AB	AC	AD	BB	BC	BD	CC	CD	BE or CE
*n*	1	1	13	6	2	11	1	38	13	3

### 3.4 Genotyping and lung maturation analysis

**FIGURE 4:**
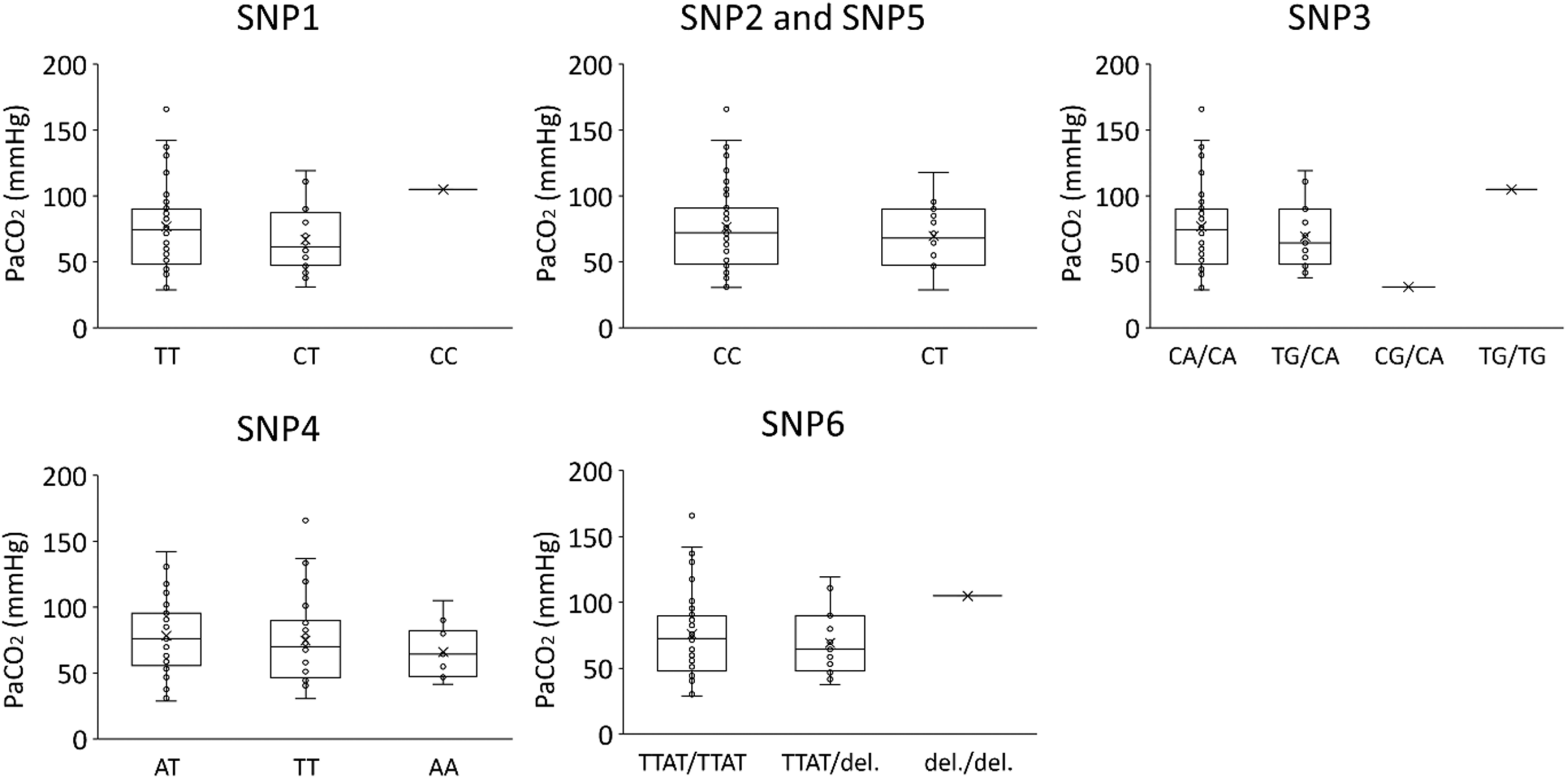
SNPs and PaCO_2_. The graphs show PaCO_2_ at 30 min of ventilation in different SNPs and alleles. There was no significant difference between alleles in each group.

**FIGURE 5:**
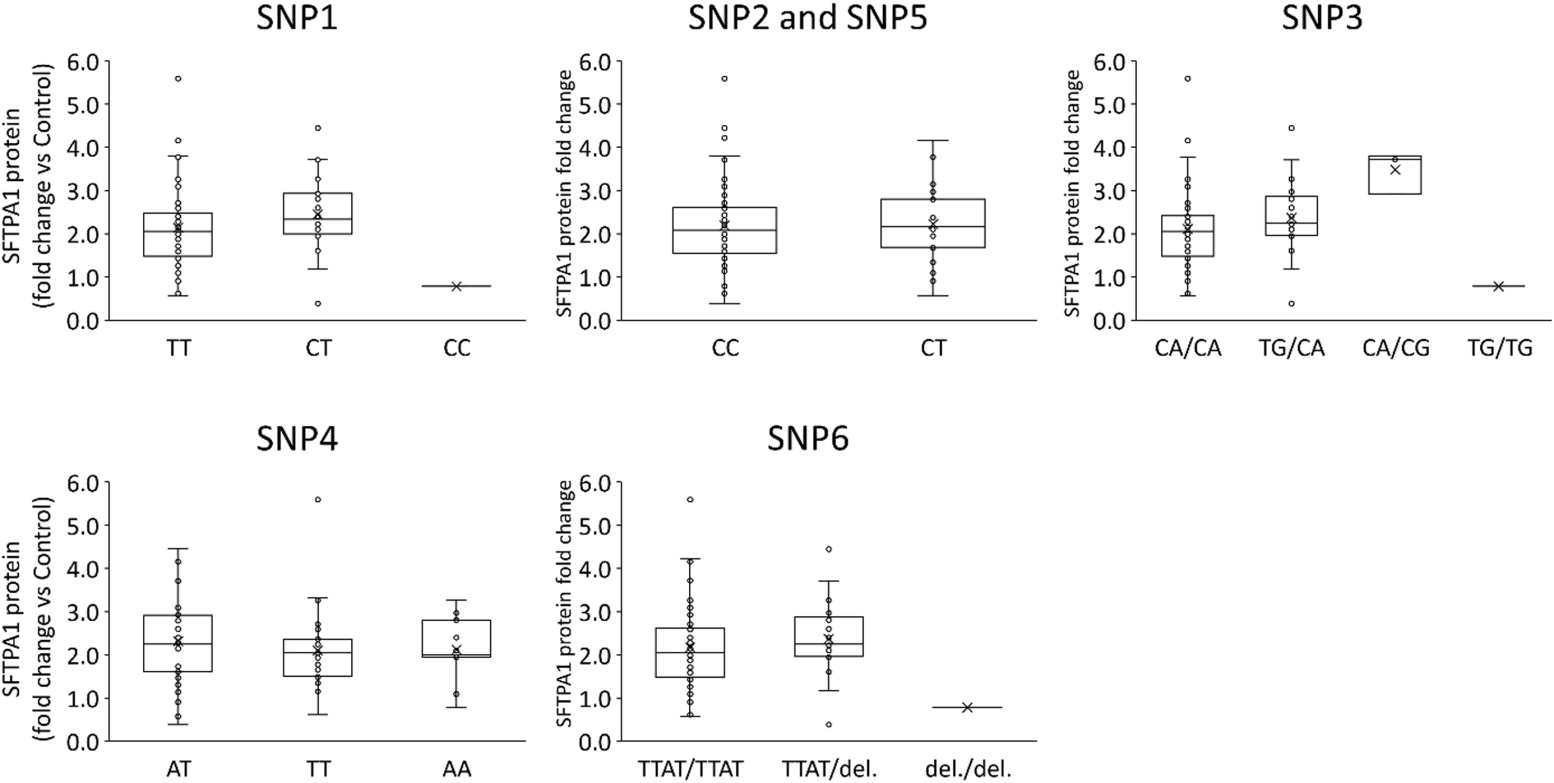
SNPs and SFTPA1. Graphs show SFTPA1 protein fold changes for different SNPs and alleles. There was no significant difference between alleles in each group.

**FIGURE 6:**
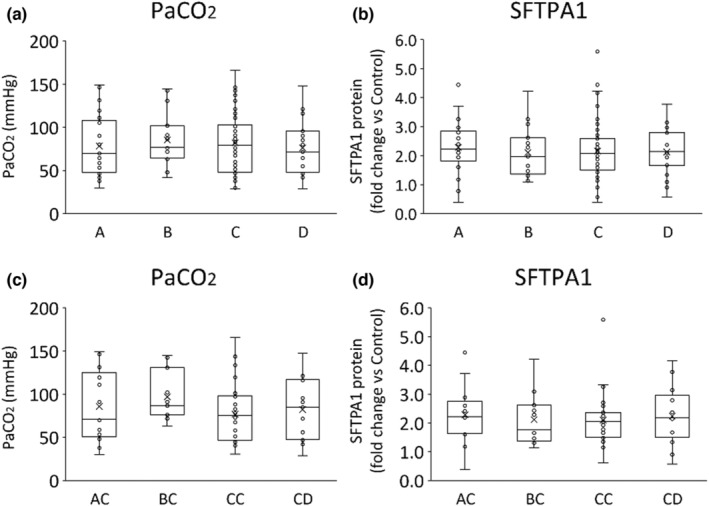
Haplotypes or diplotypes and lung maturation. (a, b) Graphs show PaCO_2_ or SFTPA1 protein fold changes for different haplotypes. There were no significant differences between haplotypes. (c, d) The graphs show PaCO_2_ or SFTPA1 protein fold changes in different diplotypes including haplotype C. There were no significant differences between diplotypes.

Each SNP, haplotype, or diplotype was compared with PaCO_2_ at 30 min of ventilation or SFTPA1 protein amount (Figures 4, 5, 6). Since most lambs had haplotype C, only lambs who had haplotype C were used in the diplotype analysis (*n* = 75). We could not detect any specific SNP, haplotype, or diplotype which was correlated to PaCO_2_ or SFTPA1 protein concentration.

## 4 COMMENTS

### 4.1 Primary findings

The primary findings of this study are as follows:

1. Six unique SNPs were detected in preterm fetal sheep undergoing ANS therapy in *Ovis aries SFTPA1* sequence of NCBI (NM_001009728.2);
2. SNPs were distributed across five haplotypes;
3. Although SFTPA1 protein concentration correlates with lung function, neither SNPs nor haplotypes correlated with either SFTPA1 protein concentration or preterm lung function following ANS treatment.

We thus concluded that, in the population of lambs in this study, polymorphisms in SFPTA1 were not associated with variability in ANS treatment efficacy. We have previously shown that although ANS therapy improves overall fetal lung maturation in the preterm sheep, around 40% of animals do not respond and retain lung function equivalent to control animals treated with saline only (Takahashi et al., 2020). We could not explain the observed response variation on the basis of mRNA expressions (*SFTPA1, SFTPB, SFTPC, AQP1, AQP5, or ENaC‐B*) or betamethasone pharmacokinetics, including the amount and duration of exposure.

### 4.2 Surfactant protein and fetal lung maturation

We previously showed that not SFTPC but SFTPA1 or SFTPB protein concentration was correlated to the fetal lung maturation in response to ANS treatments at 48 h after initial treatment when samples from saline control group animals were included (Takahashi et al., 2021). In particular, SFTPA1 protein was strongly correlated to fetal lung maturation. We also showed that neither SFTPB nor SFTPC but SFTPA1 protein and saturated phosphatidylcholine (Sat‐PC) were correlated to the fetal lung maturation at 8 days after initial treatment when samples from the saline control group and ineffectively treated groups were included (Takahashi, Takahashi, et al., 2022). In this study, we eliminated assessment of the Saline Control Group animals and ineffective ANS treated groups (to prevent type I error) and included data from additional steroid‐treated (dexamethasone) animals to increase the population of lambs studied and to assess individual responses to ANS therapy. There was no correlation between *SFTPA1* mRNA and PaCO_2_ at 30 min of ventilation in animals delivered at 48 h after initial treatment. As previously reported, since *SFTPA1* mRNA returns to baseline levels at 7 days after ANS treatment, this marker was not analyzed from 8 day treatment study animals (Tan et al., 1999). However, there was a correlation between SFTPA1 protein and PaCO_2_. This correlation was observed in both two‐day and eight‐day animals. However, the SNPs identified in this study did not correlate with preterm lung function, despite reports in clinical studies linking SFTPA1 mutations to adverse neonatal respiratory outcomes (Amatya et al., 2021; Floros et al., 2001; Kala et al., 1998; Ramet et al., 2000; Weber et al., 2000).

SFTPA1 is an attractive target for mutation screening in the setting of lung maturation. In general, lung surfactant consists of surfactant protein at 10% and lipids at 90% (Lopez‐Rodriguez & Perez‐Gil, 2014). Surfactant protein (SP) is classified into SP‐A, B, C, and D. SP‐B and SP‐C are hydrophobic surfactant proteins essential for the alveolar stability by reducing surface tension (Lopez‐Rodriguez & Perez‐Gil, 2014). SP‐A and SP‐D are hydrophilic proteins and members of the collectin family and are known traditionally as being involved in the innate immune defense system (Crouch & Wright, 2001; Johansson & Curstedt, 1997; Orgeig et al., 2010). SP‐A also interacts with phospholipids and constructs tubular myelin formation. While SP‐B deficiency by gene mutation is lethal in the first 6 months of life, SP‐A deficiency is not reported in human (Barnett et al., 2017; Magnani & Donn, 2020; Nogee et al., 1994). In animal studies it has been shown that SP‐A knock‐out mice did not have lung functional deficits (Ikegami et al., 2000). As such, in general, SP‐B and SP‐C are thought to be more important surfactant components to prevent alveolar collapse than SP‐A or SP‐D. On the contrary, some clinical reports have concluded that low SP‐A was associated with RDS and very low levels of SP‐A were observed in the lungs of babies who died from RDS, although this is not a uniform finding (Chang et al., 2016; deMello et al., 1989, 1993; Hallman et al., 1991; Moya et al., 1994). As such, SP‐A deficiency is not lethal and the necessity of SP‐A for preventing neonatal RDS in preterm born babies is controversial. The correlation between SFTPA1 protein and PaCO_2_ at 30 min of ventilation in this study supports the importance of SP‐A for preterm infants. As such, it is tempting to speculate that increasing the SFTPA1 protein level in lung is one of the factors necessary to optimize ANS therapy. At the same time, however, we should be aware that SFTPA1 protein was less correlated to the fetal lung maturation in lambs delivered 8 days after initial treatment than ones at 48 h after initial treatment. As we previously reported, Sat‐PC started to increase at 5 days after initial treatment and was strongly correlated to lung maturation in lambs who were delivered at 8 days after initial treatment (Takahashi, Takahashi, et al., 2022). Thus, although the production of SFTPA1 likely contributes to variability in lung maturation after ANS therapy, it is not a unique factor affecting fetal lung maturation.

### 4.3 Genotyping and lung maturation analysis

Since SFTPA1 protein concentration had a strong correlation with lung function, it was reasonable to look for SNPs in this gene that might alter size, structure, or transport of SFTPA1. In general, many SNPs affect the function of protein products and contribute to disease. For example, SNPs in *NR3C1*, which encodes the glucocorticoid receptor, have been well studied. These SNPs affect its sensitivity either positively or negatively against synthetic glucocorticoid treatment (Koper et al., 2014; Vandevyver et al., 2014). In the case of surfactant protein A, human *SFTPA* consists of two functional genes, namely *SFTPA1* and *SFTPA2*, which have various haplotypes. The most frequently observed are 6A, 6A^2^, 6A^3^, 6A^4^ for *SFTPA1* and 1A, 1A^0^, 1A^1^, 1A^2^, 1A^3^, 1A^5^ for *SFTPA2* (Floros et al., 2021). Some reported that 6A^2^ or 1A^0^ are positively (i.e. susceptibility) and 6A^4^ or 1A^5^ were negatively (i.e. protective) associated with neonatal RDS (Amatya et al., 2021; Floros et al., 2001; Kala et al., 1998; Ramet et al., 2000). Some SNPs exist in the 3′UTR and have the potential for quantitative differences (Floros & Fan, 2001). In this study, loci of SNPs in sheep were different from ones in human major haplotypes. We could not detect any SNPs, haplotypes, or haplogroups which were associated with variable lung maturation.

### 4.4 Strengths and limitations

In this study, animals were gestational age‐matched and subjected to tightly standardized treatments and ventilation procedures—allowing accurate identification of underlying variability of treatment responsiveness. A non‐responder rate of approximately 40% in the treatment population at 89 animals suggests that if *SFTPA1* mutations impacting ANS responsiveness do in fact exist in the sheep, then they are an uncommon cause of this phenomenon. Although the SNPs reported in this study are different loci from human SFTPA, around 76% of SFTPA protein is identical between ovis and humans and it is reasonable to assert that a polymorphism of concern in a critical region might impact function.

## 5 CONCLUSION

We found that fetal lung SFTPA1 protein concentration but not mRNA expression was significantly correlated to fetal lung maturation after ANS treatment. However, neither *SFTPA1* SNPs, haplotypes, nor haplogroups were associated with fetal lung maturation or variability of ANS treatment responsiveness.

## AUTHOR CONTRIBUTIONS

TT; conducting experiments, acquiring data, analyzing data, writing the manuscript, editing manuscript. YT; conducting experiments, editing manuscript. EF; conducting experiments, editing manuscript. HU; analyzing data, editing manuscript. LF; conducting experiments, editing manuscript. JN; conducting experiments, editing manuscript. AJ; designing research studies, writing the manuscript. MK; designing research studies, conducting experiments, writing the manuscript, editing manuscript.

## ETHICS STATEMENT

All protocols were reviewed and approved by the animal ethics committee of The University of Western Australia (RA/3/100/1636, RA/3/100/1702) and Murdoch University (R3330/21).

## FUNDING INFORMATION

This work was supported the Women and Infants Research Foundation, Cincinnati Children's Hospital Medical Centre, the Channel 7 Telethon Trust and the Stan Perron Charitable Foundation.

## CONFLICT OF INTEREST

The authors report no conflict of interest.
